# Schwann cells migrate along axons in the absence of GDNF signaling

**DOI:** 10.1186/1471-2202-13-92

**Published:** 2012-08-03

**Authors:** Stephan Heermann, Björn Spittau, Katalin Zajzon, Markus H Schwab, Kerstin Krieglstein

**Affiliations:** 1 Department of Neuroanatomy, University of Heidelberg, Heidelberg, Germany; 2 Department of Molecular Embryology Institute of Anatomy and Cell Biology, University of Freiburg, Freiburg, Germany; 3 Department of Neurogenetics, Max Planck Institute of Experimental Medicine, Göttingen, Germany; 4 FRIAS, University of Freiburg, Freiburg, Germany; 5 Current address: COS Heidelberg, INF, 230 69120, Heidelberg, Germany

**Keywords:** Schwann cell development, Migration, Proliferation, GDNF, PP2

## Abstract

**Background:**

During development neural crest derived Schwann Cell (SC) precursors migrate to nerve trunks and populate nascent nerves. Axonal ensheathment by SC is a prerequisite for normal nerve function and the integrity of myelinated as well as nonmyelinated axons. To provide adequate support functions, SC colonize entire nerves. One important prerequisite for this is their migration into distal axonal regions.

**Results:**

Here, we studied the role of Glial cell line derived neurotrophic factor (GDNF), a TGF-beta related growth factor, for SC migration. To this end we used a superior cervical ganglion (SCG) explant-SC migration assay, GDNF null mutant mouse embryos and a chemical inhibitor for GDNF signaling in combination with time-lapse imaging. We found that GDNF signaling is dispensable for SC migration along murine embryonic sympathetic axons. Furthermore, in vivo analyzes revealed that SC migration along the sciatic nerve is also not dependent on GDNF.

**Conclusions:**

In contrast to previous in vitro findings in the sciatic nerve and a SC precursor cell line, our results clearly indicate that GDNF is dispensable for embryonic SC migration. This is demonstrated for the sympathetic nervous system and also for the sciatic nerve in mouse.

## Background

Schwann cells (SC), the main glial cell type of the peripheral nervous system [1], are crucial for normal nerve function and long-term integrity of peripheral nerves [2,3]. Since SC provide support functions for myelinated as well as nonmyelinated axons, they populate not only heavily myelinated nerves (e.g. the sciatic nerve) but also largely nonmyelinated nerves (e.g. sympathetic nerves). During development, appropriate numbers of axons and SC are matched by tightly regulated proliferation as well as apoptosis [4-7]. SC migrate from nerve trunks along developing axons to distal nerve regions. Several signaling molecules have been shown to regulate SC migration. The neuronal growth factor Neuregulin (NRG) 1, acting through ErbB2/ErbB3 tyrosine kinase receptors [8], stimulates SC motility, as demonstrated in primary rat SC culture [9,10] or with an immortalized SC precursor cell line [11,12]. Studies in zebrafish revealed an essential role of NRG1 type III, ErbB2 and ErbB3 for SC migration in vivo [13,14] and we have recently shown that NRG1 type III/ ErbB signaling, via regulation of apoptosis in proximal axonal regions, is essential for SC colonization of distal sympathetic axons [7]. In addition to NRG1, also Glial Cell-line-Derived Growth Factor (GDNF), a TGF- beta related growth factor, has been suggested to control SC migration. When expressed in a layer of fibroblasts, GDNF promotes SC emigration from sciatic nerve sections. This activity depends on a non-canonical, Ret kinase independent [15] signaling pathway in which the neural cell adhesion molecule (NCAM) is employed in combination with the ligand binding GDNF family receptor alpha 1 (GFRa1) [16]. A stimulatory effect of GDNF on SC migration was also observed in several in vitro migration assays using an immortalized SC precursor cell line suggesting that GDNF (and NRG1) serve as chemotactic and chemokinetic molecules during peripheral nerve development [11]. Here, we addressed whether endogenous GDNF plays a role for SC migration along sympathetic nerves of mouse embryos. We took advantage of a SC migration assay using SCG explants [17] and time lapse imaging, which allows to study SC migration along outgrowing axons [7]. Unexpectedly, we found that SC migrate normally along sympathetic axons in the absence of GDNF signaling. Furthermore, in vivo analyzes of late embryonic sciatic nerves indicated that SC migration is not dependent on GDNF there.

## Methods

### Ethics statement

Animal work was carried out in agreement with the local ethical committees. The University of Heidelberg/ Regierungspräsidium Karlsruhe Referat 35 has approved this study (ID: T-07/10 and T-59/08)."

### Mice and tissue preparation

Time pregnancy matings of NMRI and GDNF KO mice [18] were performed overnight and the day of the vaginal plug was considered as day 0.5. Additional NMRI mice, obtained from Charles River, were mated during daytime. At embryonic day 16 (E16) to 18.5, mothers were sacrificed by cervical dislocation and the embryos were harvested by cesarean section. GDNF (Gdnf^tm1Lmgd^) mice were maintained by breeding to C57/Bl6 mice and intercrosses.

### SC migration assay, pharmacological treatment and quantification

**Figure 1:**
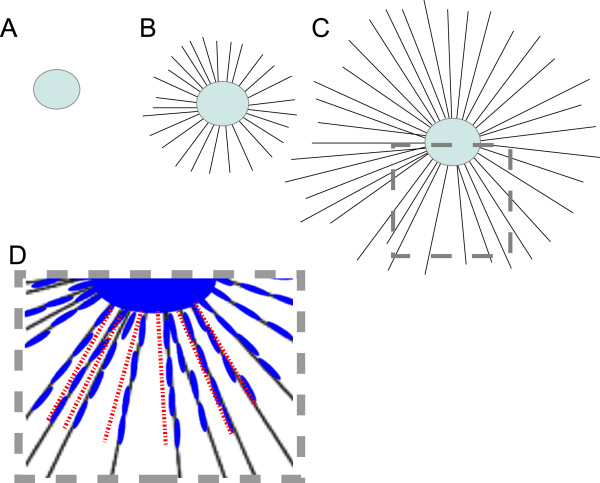
**Scheme showing the experimental setup of the SCG SC migration assay.** SCGs were explanted and put onto/in a collagen-gel. NGF treatment facilitates axonal growth (**B**) into the collagen matrix. Axons elongate further over the time (**C**) and SCs (blue) can be appreciated migrating along the axons from the SCG explant to the periphery as depicted in the close up (**D**). At the endpoint of analyses the gel was shrunk to two dimensions to enable the measurement from the border of the SCG explant to the leading SC (red dotted lines in D).

The assay used to analyze SC migration was recently described [7]. Briefly, superior cervical ganglia (SCGs) were dissected from mouse embryos at embryonic day 15.5- 18.5 (E15.5 - E18.5). After washing in dPBS SCGs were put on a three dimensional matrix of collagen. Collagen was prepared from rat-tails and the matrix was assembled according to the protocol of T. Ebendal [19]. SCGs were treated with Nerve Growth Factor (NGF, R&D or Roche, 30 ng/ml) to facilitate optimal axonal development and allow SC migration along elongating axons (Figure1). PP2 [20] a compound inhibitor for SRC-kinases and Ret kinase was used at a concentration (5.3 μM, stock concentration 53 mM in DMSO). PP2 was either added at day in vitro (DIV) 0 or at DIV3. In the latter case the assay was stopped at DIV4. Analyzes of SC proliferation were performed by measuring the pHH3/ DAPI ratio in sampled areas of 11 control and 10 PP2 treated explants. For analyzes of SC migration, distances from the explant to leading SCs of 13 control and 12 PP2 treated explanted ganglia were measured. When indicated Aphidicolin (Serva, 10 μg/μl stock in DMSO, working concentration 2 μg/ml) was added to the cultures.

### Western Blot for GDNF

Rat-tail collagen samples at different dilutions, proteins from rat adrenal gland, rat cerebrum, PC12 cells and recombinant murine GDNF (Peprotech, Hamburg, Germany) were loaded on a 13% SDS-acrylamide gel for electrophoresis. Proteins were transferred onto a PVDF membrane (Immobilon, Millipore, Schwalbach, Germany) using wet-blotting technique. After transfer, membranes were washed with TBST and blocked with 10% dry milk (Roth, Karlsruhe, Germany) in TBST for 2 h at room temperature. For detection of GDNF, membranes were incubated with primary anti-GDNF antibody (against human and rat GDNF, R&D Systems, Germany) at 4°C overnight. The primary antibody treatment was followed by treatment with HRP-conjugated goat anti-rabbit antibody (1:10,000, Cell Signaling Technologies) for 1 h at 4°C. Gapdh was used as a loading control for positive controls from rat tissue samples. Labelled proteins were detected by using Western Lightning® Plus–ECL, Enhanced Chemiluminescence Substrate (Perkin-Elmer, Germany). All blots were captured with Amersham Hyperfilm^TM^ ECL (GE Healthcare).

### Immunohistochemistry and statistical analyses

Immunohistochemical stainings for S100 (Sigma, mouse, monoclonal, 1:10-50) phospho-Histone H3 (pHH3, Milipore, rabbit polyclonal, 1: 400) Thyrosine Hydroxylase (TH, Chemicon, rabbit, polyclonal, 1:1000, Chemicon mouse, 1:400) and nuclear counterstaining with DAPI (1:10000) were performed according to standard protocols. Briefly, explant containing collagen gels were fixed with 4% PFA at the end of an experiment and directly processed for whole mount immunohistochemistry. Tissue was blocked in PBS containing 10% normal donkey serum and 2% Triton x 100 for 2 hours with consecutive antibody incubation in blocking solution over night. The next day, after washing, the tissue was incubated with labeled secondary antibodies (donkey anti rabbit/ donkey anti mouse/Alexa488/ CY3). Explants used for whole mount immunohistochemistry were dried on microscope slides, for best analyses in two dimensions and mounted in aqueous mounting medium (Mowiol). Images were taken by conventional fluorescence and non-fluorescence microscopy with Olympus and Leica microscopes respectively. Measurements were performed via the software imageJ (NIH). For statistical analyzes of quantitative data Graphpad Prism software was used.

### Time lapse imaging

Where stated, time-lapse recordings were performed in near live time temporal resolution. The recorded frame rate is 10 minutes for S3 and S4 and 30 minutes for S1, S2, S5 and S6. The scale bars represent 100 μm. For time lapse recordings a conventional inverse Microscope Setups (Leica and Nikon) connected to an incubation chamber and a heating unit was used to facilitate humid conditions with 37C° and 5% of CO2.

### Semi-thin sections

Sciatic nerves were dissected from fixed E18.5 mouse embryos. After postfixation the nerves were processed and embedded in Epon (EM-TP, Leica). Sectioning was performed with an ultramicrotom (Leica) and sections were stained with methylenblue/azurII.

## Results

### Early blockade of Src and Ret kinases disrupts axonal SC colonization

**Figure 2:**
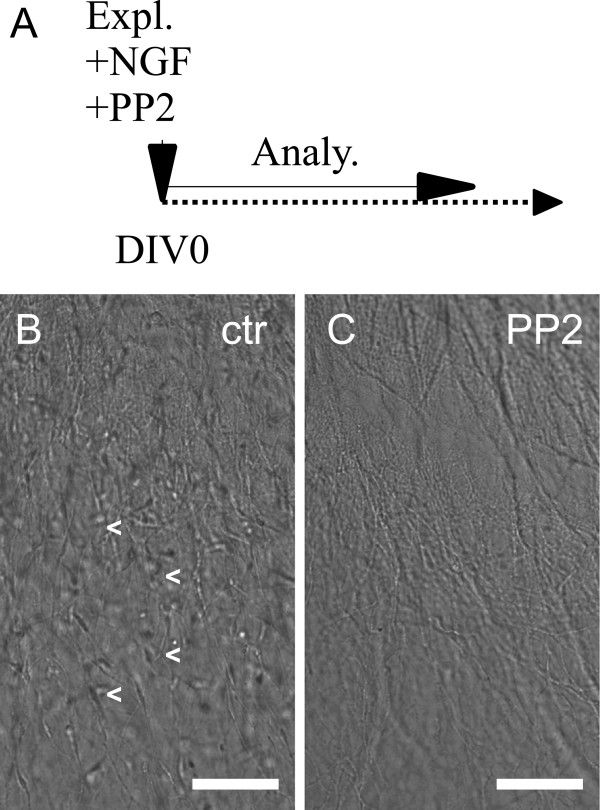
**PP2, applied at DIV0, prevents axonal SC colonization.A**: Scheme of experimental setup (Expl. = Explantation, Analy. = Analysis, DIV = Day In Vitro). NGF as well as PP2 were added at DIV0 after SCG explantation. **B**/**C**: close up of axons grown from SCG explants. Scale bars = 100 μm. Numerous SCs populating axons also in distal regions can be observed in a control explant (**B**) whereas only bare axons are visible in a PP2 treated explant (**C**).

Binding of GDNF to its GPI anchored receptor GFRa1, can induce two distinct signaling pathways. The first described was signaling via recruitment of Ret tyrosine kinases to the GDNF/ GFRa1 complex. The more recently observed pathway works via interaction with the neural cell adhesion molecule (NCAM). The latter way was described to utilize fyn a src-related kinase in the signaling cascade [15,16]. Blockade of both pathways, by inhibition of src and Ret kinases with PP2, at day in vitro (DIV) 0 (treatment scheme Figure2A) disrupted SC colonization of SCG axons (Figure2C). This can additionally be appreciated in time-lapse recordings (Additional file 1: Movies S1 and Additional file 2: S2). While SC migrate along axons in control explants (Figure2B, Additional file 1: Movie S1), migrating SC were virtually absent when explants were treated with PP2 (Additional file 1: Movie S2).

### Late blockade of Src and Ret kinases reduces SC proliferation

**Figure 3:**
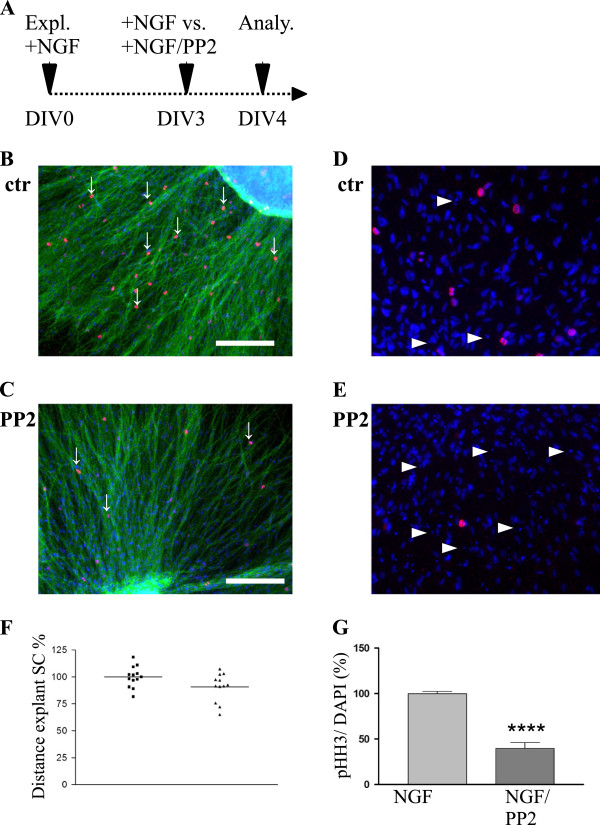
**PP2 rather acts on SC proliferation and survival than on direct SC migration.A**: Scheme showing the experimental setup (Expl. = Explantation, Analy. = Analysis, DIV = Day In Vitro). **B**/**C**: Immunohistochemical stainings for TH (green) and pHH3 (red) with DAPI nuclear counterlabeling of control (**B**) and PP2 treated (**C**) SCG explants. Scale bars = 200 μm. Please note the reduced number of pHH3 positive cells (white arrows) after PP2 treatment in comparison to the control. D/E: close up of ctr (**D**) and PP2 treated (**E**) SCs costained with DAPI nuclear labeling and pHH3. One can appreciate the increased level of fragmented nuclei after PP2 treatment. **F**: Quantitative analyzes of SC migration distances in %. (students *t* test: p value = 0.0508) No significant difference in migration distances was observed when PP2 was added at DIV3. **G**: Quantitative analyzes of Proliferation using the pHH3/DAPI ratio in %. A strongly reduced level of mitosis was observed, when PP2 was added at DIV3 (students *t* test: p value < 0.0001).

To directly address the effect of Ret and src kinase signaling inhibition on SC migration, PP2 was administered at DIV3 (treatment scheme Figure3A), when numerous axon-associated SC already migrated away from the NGF treated explants. Migration distances from the SCG explant to the leading SC were measured at DIV4 (Figure1D). Unexpectedly, under these conditions only a small (not significant) reduction in SC migration distances was observed (Figure3F). Additional time-lapse recordings (Additional file 3: Movie S3 ctr and Additional file 4: Movie S4 PP2) show migrating SC in the PP2 treated sample similar to the control. These findings argue against a direct role of Src or Ret kinases in SC migration along sympathetic axons. However, we want to note that SC motility seems by trend slightly reduced after PP2 treatment (Additional file 3: Movie S3/Additional file 4: Movie S4).

If a SCG explant is solely treated with NGF, large numbers of SC migrate away from the explant to distal axonal regions (Additional file 1: Movie S1). We speculate that SC precursors must massively proliferate before the onset of migration. To test whether PP2 affects SC proliferation we determined pHH3/DAPI ratios of SCG explants treated with PP2 at DIV3. We observed a reduction in the number of proliferating SC in PP2 treated samples compared to controls (Figure3G). In addition more fragmented SC nuclei were visible after PP2 treatment, in line with increased SC apoptosis (Figure3D/E). This suggests that the failure in colonization of sympathetic axons after PP2 treatment at DIV0 might result from reduced numbers of premigratory SC in the explants.

### Blocking SC proliferation at DIV0 recapitulates early PP2 phenotype

**Figure 4:**
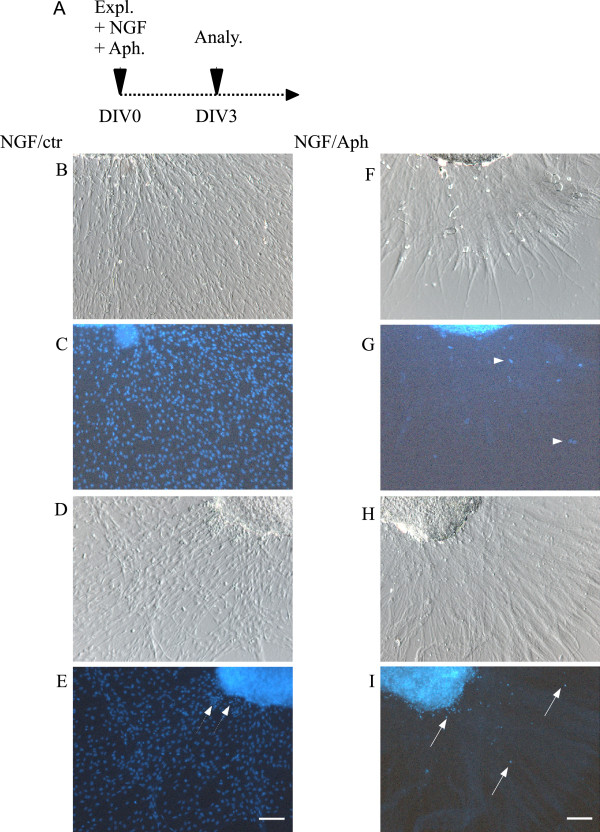
**Early inhibition of SC proliferation also prevents axonal SC colonization.A**: Scheme showing the experimental setup (Expl. = Explantation, Aph. = Aphidicolin Analy. = Analysis, DIV = Day In Vitro). **B**,**D**,**F** and **H** show brightfield images of axonal growth of control (**B** and **D**) and aphidicolin treated samples (**F** and **H**). In both conditions axonal growth could be observed. **C**, **E**, **G** and I show DAPI stainings of controls (**C** and **E**) and aphidicolin treated samples (**G** and **I**). Where in the control condition a multitude of SC could be observed, in the aphidicolin treated sample only rarely cells could be observed outside the ganglion (arrows). Nuclear detritus was observed in both conditions (arrowheads). (Scale bars = 100 μm).

To test our hypothesis that reduced SC proliferation disrupts axonal SC colonization, we blocked SC proliferation at DIV0 by treatment with aphidicolin, a DNA polymerase inhibitor [7], (treatment scheme Figure4A) and analyzed SC migration. In controls as well as after aphidicolin treatment axonal growth was observed (Figure4B/F/D/H). However, hardly any SC were detectable that migrated out of the explant along the axons after SC proliferation was inhibited (Figure4G/I). Only occasional cells can be observed along the elongated axons (Figure4, arrows). In addition to blocked proliferation, we also noticed nuclear debris (Figure4, arrowheads) in controls and aphidicolin treated explants. Cells either died along axons or the detritus was pushed away from the explants during axonal growth. The almost total lack of SC after blockade of SC proliferation at DIV0 suggests that a certain amount of SC is required to initiate migration.

### GDNF is dispensable for SC migration along sympathetic axons

**Figure 5:**
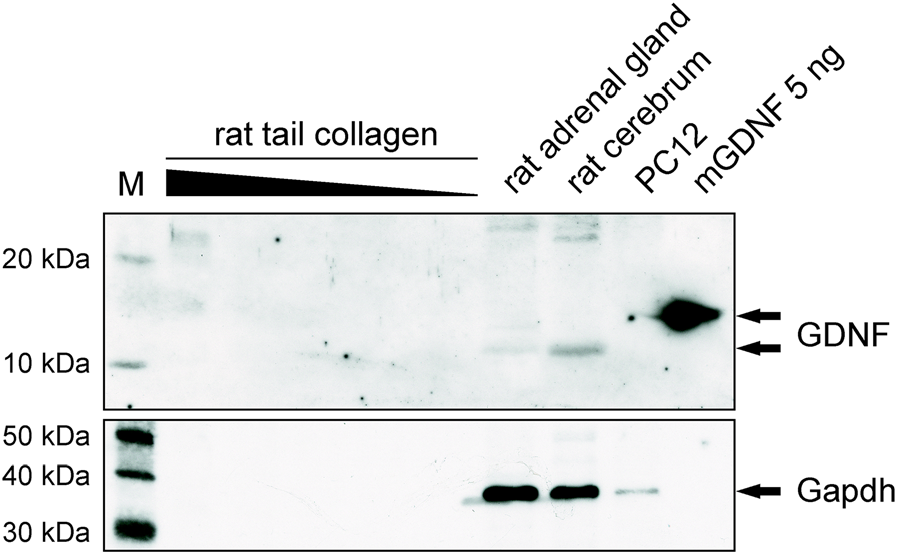
**The SCG SC development assay is free of GDNF.** Western Blot for GDNF with different dilutions of rat-tail collagen (20μl, 10μl, 5μl, 2.5μl, 1.25μl and 0.6125μl collagen) were analyzed using anti-GDNF antibody specific for human and rat GDNF. Rat adrenal gland (20μg), rat cerebrum (20μg), proteins from undifferentiated PC12 cells (20μg) and recombinant murine GDNF (5ng) were used as positive controls. Gapdh was used a loading control for tissue samples. Whereas recombinant murine GDNF as well as rat GDNF in samples from adrenal gland and cerebrum were detectable, no GDNF signal was obtained in the rat-tail collagen of any amount analyzed.

Next we directly addressed the role of GDNF for SC migration along sympathetic axons by using SCG explants from GDNF knock out (KO) mice [18]. To rule out possible GDNF-contamination of the 3D collagen (rat-tail) matrix based migration assay we performed a Western Blot analyzing various amounts of rat-tail collagen. As positive controls commercially available murine GDNF and protein samples from rat adrenal gland, rat cerebrum as well as PC12 cells were used. While GDNF monomers were readily detected in the rat adrenal gland and rat cerebrum, no GDNF signals were detectable in any of the collagen dilutions (Figure5).

**Figure 6:**
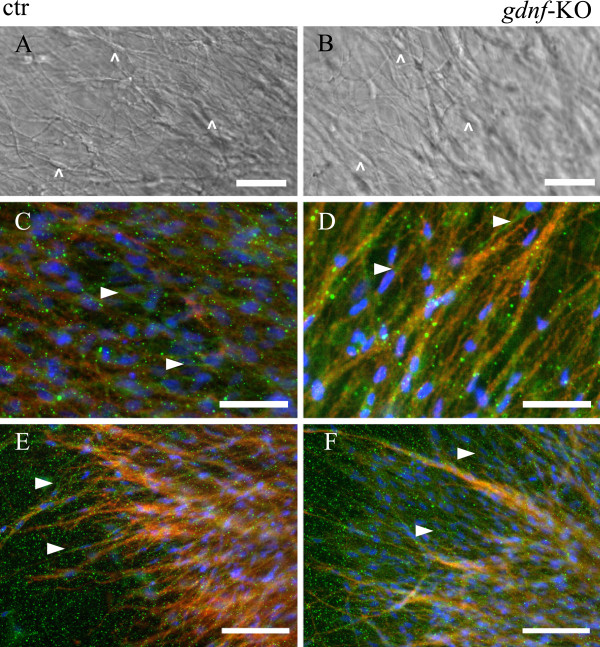
**GDNF is dispensable for SC migration along embryonic sympathetic axons.A**/**B**: bright field images taken during time-lapse imaging (scale bar 100 μm). **A**: control SCG explant treated with NGF. A multitude of axons are visible and SCs along the axons (arrowheads). **B**: SCG explant of GDNF KO. Also many axons are visible, surprisingly also covered by SCs (arrowheads). **C**-**F**: Immunohistochemical stainings (scale bar 200 μm), Thyrosine hydroxylase (TH, red) and s100 (green) immunostainings with DAPI nuclear counterstaining. In both control (**C**/**E**) as well as in the GDNF KO explants (**D**/**F**) many sympathetic axons labeled by TH can be seen, covered with s100-ir positive labeled SCs. **C**/**D**: E18.5, **E**/**F**: E15.5.

Having demonstrated that collagen gels are free of GDNF, we took advantage of the migration assay and analyzed GDNF- deficient SCG explants in comparison to controls. In GDNF KO as well as control SCG explants a multitude of sympathetic axons were observed growing from the explanted ganglia visible in bright field as well as after TH-immunohistochemistry (Figure6). This indicates that the overall axonal growth is not affected by the loss of GDNF in SCG explants. Unexpectedly however, also many S100 positive cells were observed associated with the axons in both control as well as GDNF mutant explants in proximal and also more distal axonal areas. In addition time-lapse movies (Additional file 5: Movie S5 and Additional file 6: Movie S6) demonstrae unaltered axonal growth and normally migrating SC along elongating axons in both control and GDNF mutant explants. Together, these data demonstrate that GDNF is not required for SC migration along sympathetic axons.

### GDNF is also dispensable for SC colonization of the sciatic nerve

**Figure 7:**
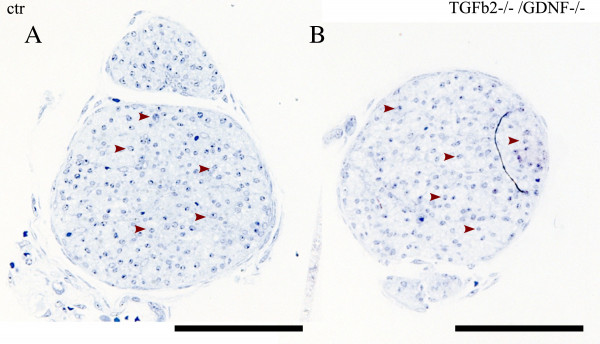
**GDNF is dispensable for SC migration along the embryonic sciatic nerve.A** and **B** show orthogonal semithin sections of sciatic nerves of a control (**A**) and a TGFb2/GDNF double mutant (**B**). Both nerves show a multitude of SC (arrowheads). (Scale bar = 100 μm).

Next we addressed in whether GDNF affects SC colonization of other peripheral nerves. To this end we examined orthogonal semi-thin sections of GDNF/TGFb2 double mutant sciatic nerves and controls at E18.5. Due to perinatal death of the GDNF mutant mice the analyses are restricted to embryonic stages [18]. It was previously shown that TGFb facilitates GDNF signaling by recruiting GFRa1 to plasma membranes [21]. This makes the TGFb2/GDNF double mutant a good model to study GDNF function. However, no differences were observed between the control and the double mutant sciatic nerves (Figure7 A/B). In both cases a multitude of SC can be observed in the orthogonal sectioned nerve (Figure7 arrowheads). This demonstrates that, at least during embryonic stages, GDNF is dispensable for SC migration in peripheral nerves.

## Discussion

SC are a fundamental component of peripheral nerves and either solely ensheath or additionally myelinate axons. By both, SC support the long-term integrity of axons with the latter also rapid impulse propagation [22]. SC development, including proliferation, survival, migration and myelination is regulated by axonal signals [23]. Many studies have addressed the molecular processes that control SC development and disease, however most of these focused on myelination ([3,24], Svaren and Meijer, 2008). Less is known about the signals that regulate SC colonization of axons following the migration of SC precursors from the neural crest to nerve trunks [25]. GDNF was previously observed to promote migration of immortalized SC precursors and primary sciatic nerve SC [11,16]. SC express GFRa1 the GDNF binding receptor [26,27] in line with the hypothesis that GDNF can directly signal to SC. To test the role of GDNF for SC migration along sympathetic axons we used a SC migration assay, containing elongating axons, the physiological substrate for SC precursor migration [7], a compound chemical inhibitor (PP2, [20]) for Src as well as Ret kinases, signaling cascade components of non-canonical and canonical GDNF signaling respectively, and SCG explants of embryonic GDNF KO mice [18] in combination with time lapse imaging.

Application of PP2 at DIV0 inhibited axonal SC colonization, so only bare axons were visible during time-lapse imaging. Further analyzes however, focusing on SC which were already migrating along growing axons (DIV3) showed that PP2 only had a mild and insignificant effect on SC migration distances. However, PP2 was observed to reduce SC proliferation and increase SC apoptosis. By blocking SC proliferation (with Aphidicolin) at DIV0 we were also able to sufficiently block axonal SC colonization. We therefore concluded that PP2, if applied at DIV0, indirectly inhibited axonal SC colonization, by reducing the number of SC precursors. Though, even if the phenotype induced by PP2 at DIV0 was caused indirectly it was severe and could eventually support the hypothesis that GDNF is crucial for axonal SC colonization. Importantly however, in contrast to our expectations, we found no obvious SC migration phenotype, analyzing SCG explants of GDNF null embryos in comparison to controls. Accordingly, we had to conclude that GDNF signaling is neither absolutely required for the proliferation of premigratory SC nor for their subsequent migration along sympathetic axons. The absence of obvious defects in SC development also indicates that GDNF- mediated axonal release of heparin-binding forms of NRG1 as observed for cultured DRG neurons [28] is not essential for the development of SC in the sympathetic nervous system or is compensated by the in excess available NGF, which is also able to release heparin binding forms of NRG1 [28]. The different outcomes of our study and the previous findings could result from different model systems used, e.g. that GDNF may serve distinct functions in myelinated and nonmyelinated nerves. However, nonmyelinating SC have been shown to respond to (exogenously added) GDNF, which stimulates SC proliferation and myelination of small caliber axons in adult rats [29]. Finally we wanted to address the in vivo role of GDNF for SC migration. As GDNF KO mice are not viable [18], analyzes were restricted to embryonic stages in which the dissection of postganglionic SCG nerves is hardly feasible. Therefore we decided to analyze late embryonic (E18.5) sciatic nerves of GDNF mutants in comparison to controls. In line with our previous results in the sympathetic nervous system also in the sciatic nerve of GDNF (GDNF/ TGFb2) mutants in comparison to controls no difference with respect to SC populations could be observed. This indicates that GDNF is dispensable for embryonic SC migration in vivo. Even though endogenous GDNF is dispensable for normal SC development it cannot be ruled out that exogenously introduced GDNF is able to affect SC migration, which could explain previous findings ([16], Corenjo et al., 2010). Alternatively, other GDNF family ligands (such as Neurturin) may compensate for the loss of GDNF in SCG explant cultures and in sciatic nerves in vivo. Normal SC development in GDNF deficient SCG explants in contrast to massively reduced SC proliferation after PP2 application indicates that Src and/ or Ret kinase activities in SCG explants are regulated by additional extracellular signals. NRG1 signaling regulates SC proliferation [30,31], and we have recently shown that NRG1 type III-ErbB signaling promotes SC colonization of distal sympathetic regions by preventing apoptosis in proximal regions [7]. Since NRG1 signaling also stimulates Src kinase activity [32,33], it is plausible that Src inactivation by PP2 mimics to some extend the loss of NRG1 type III ErbB signaling. Though, it is interesting to note that blocking ErbB receptors leads next to reduced SC proliferation also to reduced SC colonization of distal axonal compartments [7], together indicating a more complex ErbB downstream signaling. Taken together we demonstrate that SC can migrate along sympathetic axons an sciatic nerve axons of GDNF deficient embryos leading to the conclusion that GDNF as a factor and also GDNF signaling is dispensable for SC migration along murine embryonic axons.

## Conclusions

In this study we analyzed the role of GDNF for embryonic SC migration. In contrast to previous in vitro findings in the sciatic nerve and a SC precursor cell line [11,16], our data clearly indicate, that GDNF is dispensable for embryonic SC migration along sympathetic axons, demonstrated with the SCG explant SC development assay, as well as along the sciatic nerve, demonstrated with the help of semithin sections of mutant nerves. Although PP2, a pharmacological inhibitor for canonical as well as alternative GDNF signaling, showed a strong effect on axonal SC colonization (applied at DIV0 to the SCG explant assay), this effect is in fact independent of GDNF. This is clearly shown, as no phenotype could be observed when GDNF mutant SCGs were used for the SCG explant SC migration assay (Figure6 and Additional file 5: Movie S5 and Additional file 6:Movie S6). Further investigations revealed that PP2 acts rather on SC proliferation and on SC survival than on SC migration directly (Figure3). SC motility, was only affected by trend shown in the quantitative analysis (Figure3F) as well as in the Additional file 3: Movie S3/Additional file 4: Movie S4. These data suggest that SC proliferation is a prerequisite for the initiation of SC migration, underlined by the fact that early blockade of SC proliferaion also prevented axonal SC colonization (Figure4). The phenotype induced by PP2, however, must be the result of an alteration of a different signaling pathway. As Neuregulin1/ErbB signaling can also act via a src kinase [32,33] and was shown to be involved in SC proliferation [7,30,31] we conclude that PP2 is interfering with this pathway. Altogether we demonstrated that SC can migrate along axons in the absence of GDNF signaling.

## Competing interests

The authors have no financial or non-financial competing interests.

## Authors’ contributions

SH conceived the study, carried out the SCG assays, performed the semithin sectioning, performed time-lapse-imaging and wrote the manuscript. BS performed the western blots and wrote the according sections of the manuscript. KZ participated writing the manuscript. MHS participated in drafting and writing the manuscript and gave important scientific input. KK conceived the study and helped writing the manuscript. All authors read and approved the final manuscript.

## Supplementary Material

Additional file 1 **Supplemental time-lapse movies (scale bars = 100 μm).**Time-lapse movie S1 Ctr SCG Explant (treatment DIV0) (Imaging started DIV1, frame rate: 30 min). Click here for file

Additional file 2 Time-lapse movie S2 PP2 treated SCG Explant (treatment DIV0) (Imaging started DIV1, frame rate: 30 min). Click here for file

Additional file 3 Time-lapse movie S3 Ctr SCG Explant (treatment DIV3, Imaging started DIV3, frame rate: 10 min). Click here for file

Additional file 4 Time-lapse movie S4 PP2 treated SCG Explant (treatment DIV3, Imaging started DIV3, frame rate: 10 min). Click here for file

Additional file 5 Time-lapse movie S5 Control SCG Explant (Imaging started DIV4, frame rate: 30 min). Click here for file

Additional file 6 Time-lapse movie S6 GDNF mutant SCG Explant (Imaging started DIV4, frame rate: 30 min). Click here for file
